# Herbivory Damage Increased VOCs in Wild Relatives of Murtilla Plants Compared to Their First Offspring

**DOI:** 10.3390/metabo13050616

**Published:** 2023-04-30

**Authors:** Manuel Chacón-Fuentes, Leonardo Bardehle, Ivette Seguel, Javier Espinoza, Marcelo Lizama, Andrés Quiroz

**Affiliations:** 1 Agriaquaculture Nutritional Genomic Center, CGNA, Temuco 4780000, Chile; 2 Laboratorio de Química Ecológica, Departamento de Ciencias Químicas y Recursos Naturales, Universidad de La Frontera, Av. Francisco Salazar 01145, Casilla 54-D, Temuco 4811230, Chile; leonardo.bardehle@ufrontera.cl (L.B.);; 3 Centro de Investigación Biotecnológica Aplicada al Medio Ambiente (CIBAMA), Universidad de La Frontera, Av. Francisco Salazar 01145, Casilla 54-D, Temuco 4780000, Chile; 4 Departamento de Producción Agropecuaria, Facultad de Ciencias Agropecuarias y Medioambiente, Universidad de La Frontera, Av. Francisco Salazar 01145, Casilla 54-D, Temuco 4811230, Chile; 5 Innovalimentos SPA, Gabriela Mistral 02311, Temuco 4780000, Chile; 6 Programa de Doctorado en Ciencias Agroalimentarias y Medioambiente, Facultad de Ciencias Agropecuarias y Medioambiente, Universidad de La Frontera, Temuco 4811230, Chile

**Keywords:** murtilla, mechanical damage, herbivory damage, VOCs

## Abstract

Murtilla (*Ugni molinae*) is a shrub native to Chile that has undergone an incipient domestication process aimed at increasing its productivity. The reduction in intrinsic chemical defenses due to the domestication process has resulted in a decrease in the plant’s ability to defend itself against mechanical or insect damage. In response to this damage, plants release volatile organic compounds (VOCs) as a means of defense. To understand how domestication has impacted the production of VOCs in the first offspring of murtilla, we hypothesized that their levels would be reduced due to the induction of mechanical and herbivore damage. To test this hypothesis, we collected VOCs from four offspring ecotypes and three wild relatives of murtilla. We induced mechanical and herbivore damage in the plants and then enclosed them in a glass chamber, where we captured the VOCs. We identified 12 compounds using GC-MS. Our results showed that wild relative ecotypes had a higher VOC release rate of 624.6 µg/cm^2^/day. Herbivore damage was the treatment that produced the highest release of VOCs, with 439.3 µg/cm^2^/day in wild relatives. These findings suggest that herbivory triggers defenses through the emission of VOCs, and that domestication has influenced the production of these compounds in murtilla. Overall, this study contributes to bridging the gap in the incipient domestication history of murtilla and highlights the importance of considering the impact of domestication on a plant’s chemical defenses.

## 1. Introduction

*Ugni molinae* Turcz, known as “murtilla”, “mutilla” or “murta”, is a native shrub that inhabits the area between the coastal zone and the pre-Andean mountains in Chile [1,2]. The plant has antioxidant properties, and native people have used extracts of leaves for health benefits and consumed its fruits, and used the fruits to make alcoholic beverages and jams [3]. Over the past 25 years, murtilla cultivars have been selected mainly based on their productive traits, such as yield (kg of fruits/plant) or fruit diameter, and secondarily for their antioxidant activity [4,5,6,7,8]. These domestication efforts have led to an increase in yield and other desirable traits, but have also resulted in a reduction in the intrinsic chemical defenses of the plant. Studies have been conducted to evaluate the content of polyphenolic compounds in various parts of murtilla, including leaves, fruits, and stems [3,7,9]. These compounds have also been studied in the context of domestication, which has been found to lead to a reduction in their concentrations in cultivated plants when compared to their wild relatives. This reduction has been observed for various polyphenolic compounds, such as rutin, quercetin, or kaempferol [9,10,11,12,13]. For instance, rutin, quercetin, and kaempferol decreased their presence in cultivated plants by 17.61%, 17.50%, and 13.06%, respectively [9]. Despite some studies on the polyphenolic compounds in *U. molinae*, there is still limited research on its volatile organic compounds (VOCs) [14,15]. In fact, the study of VOCs related to domestication in *U. molinae* plants was only recently conducted by Chacón-Fuentes et al. [15]. The authors observed a quantitative reduction in the VOCs profile in cultivated ecotypes compared to their wild relatives; some compounds, such as 2-hexanone, 1,8-cineole, and *trans*-caryophyllene, were not present in the cultivated ecotypes. The emission of VOCs in plants is significant due to its association with various strategies in the regulation of pest interactions. The domestication of *U. molinae* has led to a decrease in the production of defensive compounds, which can be advantageous for repelling insect pests. This is consistent with the plant domestication defense theory, which suggests that domestication of plants leads to a reduction in intrinsic chemical defenses [16,17,18,19,20,21]. However, these compounds increase in concentration when plants are damaged, indicating that they still play a vital role in plant defense mechanisms. Hence, plants release VOCs when herbivores feed on them, which can serve as signals to predator or parasitoid insects [22,23,24,25,26]. The domestication of plants can lead to a reduction in defensive compounds that would otherwise help the plants deal with insect pests. For example, both wild and domesticated maize plants did not release *β*-myrcene when undamaged, but when exposed to feeding by *Spodoptera frugiperda*, *β*-myrcene levels increased in both types of plants [27]. In the case of murtilla plants, Chacón-Fuentes et al. [15] identified several VOCs, including 2-hexanone, α-pinene, 1,8-cineole, and *trans*-caryophyllene. Domestication of murtilla plants led to a decrease in the abundance of these compounds, but mechanical damage to the plants increased the release of these VOCs.

Plant domestication and selective breeding have been studied as some of the most relevant events in human history. Human civilization exerted selection pressure on wild crops based on their useful traits, such as shape, size, production, and fruit quality [28]. However, these useful traits and the increase in production modified the balance between growth, reproduction, and defense in cultivated plants. Thus, the selection of cultivated plants with higher productivity traits resulted in a decrease in their allocation to chemical defense, according to some studies [29,30]. This reduction in chemical defense was associated with an increase in insect abundance compared to their wild relatives, as proposed by the “plant domestication defense theory” [31,32].

Studies on the incipient domestication process in *Ugni molinae* have reported a reduction in the content of polyphenolic compounds in cultivated plants compared to their wild relatives, supporting the plant domestication defense theory. For instance, the first report on the comparison between cultivated and wild relatives of *U. molinae* was conducted in two locations (Experimental Station INIA Carillanca, Tranapuente, Chile and wild sites), which raised concerns about the influence of environmental factors on the results [9]. To address this issue, a second experiment was conducted in a common garden to eliminate the influence of location or environmental factors. This study aimed to evaluate the impact of domestication on the “plant domestication defense theory” in *U. molinae* [11]. In this report, a reduction of 21.9% and 9.7% in the content of myricetin and quercetin, respectively, was observed in cultivated plants. Additionally, a reduction in polyphenol concentration was related to an increase in the number of insects and the damage index by herbivores in cultivated plants of *U. molinae*. For example, Chacón-Fuentes et al. [10] reported that cultivated plants increased the number of insects by 50% in relation to wild relatives, and the damage index by insects increased almost threefold compared to their wild counterparts. Thus, in both cases (with the influence of location and in the common garden), the reduction of polyphenolic compounds in cultivated plants was reported, supporting the plant domestication defense theory. However, to date, all the comparisons and studies related to the incipient domestication process in *U. molinae* have been developed with clones obtained from cutting the respective wild material.

The present research is the first study to compare the VOC profile between wild relatives and the first offspring of murtilla (FOM) in *U. molinae* plants. By subjecting the murtilla plants to mechanical and herbivory damage, we evaluated VOC emission in three wild relatives and their FOM. We propose the following hypotheses: VOCs in the first offspring of murtilla are reduced due to the induction of mechanical and herbivory damage. This study will contribute to elucidating the effect of domestication on induced plant VOCs in *U. molinae*, providing valuable insights into the process of domestication and its impact on the chemical defense mechanisms of plants.

## 2. Material and Methods

### 2.1. Plant and Insect Material

Three wild relatives and four ecotypes of the FOM plants were obtained from a common garden established in Temuco, La Araucanía region, Chile. The plants were six years old and one meter in height. For the experiments, they were transported to the Laboratory of Chemical Ecology at the Universidad de La Frontera, La Araucanía region, Chile, and acclimatized at 25 °C before the experiments. To compare the VOC profile from branches, the ancestor ecotypes of *U. molinae* 19-1, 22-1, and 23-2 were grouped in the category of “wild relatives,” and the ecotypes 10-1, 16-16, 17-4, and 66-2 obtained from crosses of wild relatives were grouped in the category of “FOM” (Table 1). For the experiment, we will be using branches that are 30 cm in length. We visually inspected all branches from both categories and found them to be healthy with no visible damage, except for the damage inflicted for the study. To obtain adjusted VOC values, we standardized the branches and made adjustments based on the leaf surface area and stem diameter of each evaluated branch. To calculate the leaf area and stem surface area of the murtilla branches, all the leaves of each branch were scanned using the ImageJ 1.42 J program (U.S. National Institutes of Health, Bethesda, MD, USA). The formula for a cylinder was used to calculate the stem surface area. This allowed us to obtain the total surface area associated with the emission of VOCs from the branches of each plant [15]. For the collection of aphids, they were collected from roses located in Temuco, Region of La Araucanía, Chile. These aphids were used as parents for breeding a second generation, which was used in herbivory damage treatments in murtilla plants (see below). The aphids were reared on new shoots of murtilla plants at room temperature under laboratory conditions (25 °C). Once they reached their adult stage, the aphids were arranged with the help of a brush and placed on leaves of murtilla.

### 2.2. Damage Induction

Each of the seven ecotypes, which were grouped into wild relatives and FOM, were subjected to three different conditions. The first condition served as the control treatment, in which one branch of *U. molinae* plants was sprayed with 1 mL of distilled water with 1% acetone, 48 and 24 h prior to the VOC collection [33]. For the second condition, we established herbivory damage by depositing 10 adults of *Myzus persicae* (Hemiptera: Aphididae) on new shoots of a branch of *U. molinae* plants 48 h before the collection of VOCs. Prior to the collection of VOCs, all aphids were removed from each plant using a brush [33]. Finally, a mechanical damage treatment was performed involving cutting 10 leaves in half with scissors to simulate damage caused by a chewing insect. Additionally, 20 punctures were made on the respective stem to simulate damage caused by a sucking insect on one branch of murtilla. The first 10 punctures were made 48 h before the collection of VOCs, and the last 10 punctures were made 24 h before the collection of VOCs [33]. All experiments were replicated six times (*n* = 6).

### 2.3. Volatiles Collection System

Volatile organic compounds were collected from individual branches of each ecotype (subjected to either mechanical or herbivore damage) and control treatment over a 24-h period at a flow rate of 1 L/min. Collection was carried out using a 900 mL glass chamber (6 cm ID and 30 cm high) to enclose each branch. Volatile compounds released from *U. molinae* were absorbed on 100 mg of Porapak-Q columns (80–100 mesh; Water Associates, Bakersfield, CA, USA) that were previously cleaned with 1 mL of diethyl ether (GC grade; Merck, Darmstadt, Germany) and conditioned at 150 °C for 2 h in a nitrogen stream (70 mL/min). The entrainment was performed using a positive/negative pressure air system, according to the methodology proposed by Barrios San Martín et al. [34]. The air was dried and purified by passing through activated 5-Å molecular sieves, then charcoal, and finally drawn through the glass chamber. Volatiles were extracted from the Porapak-Q by elution with 1 mL of hexane (GC-MS grade; Optima Scientific, Darmstadt, Germany), which was concentrated to 100 μL by a nitrogen flow [15].

### 2.4. Gas Chromatography Coupled to Mass Spectrometer Analysis

VOCs were analyzed using gas chromatography coupled to mass spectrometry (GC-MS) (Focus DSQ, Thermo Electron Corporation, Waltham, MA, USA). Separation was performed using a capillary column BP-1 (30 m × 22 mm × 0.25 μm) and helium gas as the carrier (1.0 mL/min) at an initial temperature of 40 °C for 2 min, increased to 250 °C with 5 °C increments per minute. Both injector and interface temperatures were kept at 250 °C, and the detector temperature was fixed at 200 °C. The electron impact ionization energy was set at 70 eV. The acquisition of each mass spectrum was carried out in the mass range from 30 to 350 *m*/*z*. A total of 1 μL aliquot from all seven ecotypes subjected to several types of damage was injected into the GC-MS for volatile compound analysis. The experimental mass spectrum was compared with those in the NIST library (Mass Spectral Library Version 2.0), using a matching algorithm with a reverse search technique to verify the highest peaks from the reference compound. Additionally, Kovats Indexes (KIs) were determined by the injection of an alkane series (C6-C26). Furthermore, experimental KIs were compared to theoretical KIs reported in the NIST database [35]. To construct calibration curves for volatile compounds that were present in wild relatives and FOM plant samples, standard solutions were dissolved in hexane (Sigma-Aldrich, St. Louis, MO, USA) at 1000 mg/L. The stock solutions of each standard were used to prepare a serial concentration between 0.05 and 500 mg/L [36]. For the analysis of the chromatographic signals, a detection limit of 3 times the noise was used, and the limit of quantification was 10 times the noise.

### 2.5. Statistical Analysis

The statistical software Statistix 10 (Tallahassee, FL, USA) was used to perform the analysis. To compare the total VOC concentration between wild relatives and FOM in murtilla plants, a *t*-test was performed. Additionally, an analysis of variance (ANOVA) followed by Tukey’s test was carried out to compare the effect of damage treatments and domestication on the total concentration of VOCs, ecotypes, and damage treatments. Similarly, ANOVA followed by Tukey’s test was performed to analyze the total VOCs, GLVs, and terpenes concentration. Finally, to evaluate the effect of domestication and damage treatments on specific compounds, an ANOVA followed by Tukey’s test was carried out. All data were assessed for normality and homoscedasticity of variance. Values of *p* ≤ 0.05 were considered significant. Results are expressed as means and their corresponding standard errors.

## 3. Results

### 3.1. Total VOCs Identification

GC-MS analysis identified 12 VOCs, which were grouped as follows: (1) alcohols and ketones, (2) esters, (3) monoterpenes, and (4) sesquiterpenes (Table 2). Moreover, we grouped these VOCs into two categories: (1) green leaf volatiles (GLVs), which included alcohols, ketones, and esters, and (2) terpenes, which included monoterpenes and sesquiterpenes.

### 3.2. VOCs Identification Related to the Domestication Degree

**Figure 1:**
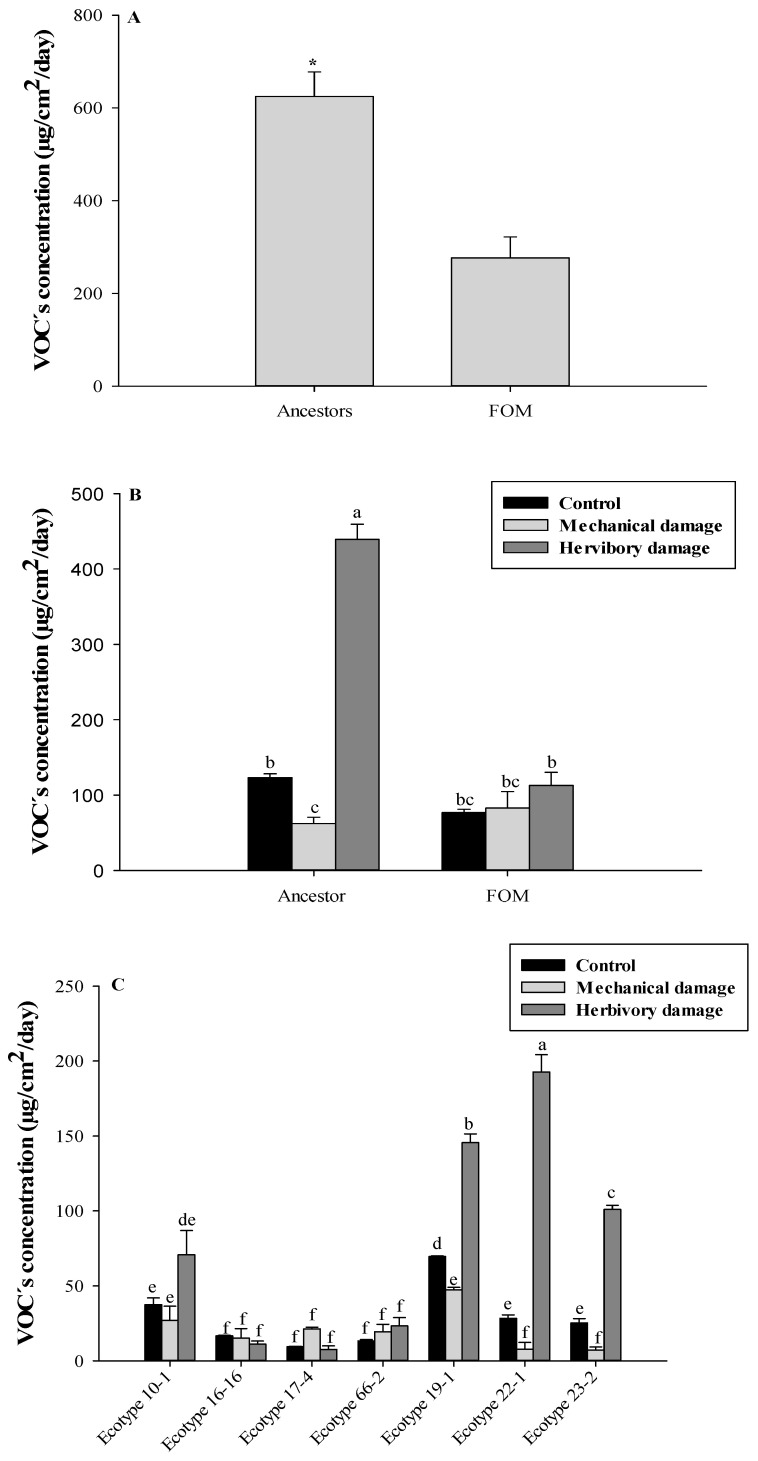
(**A**) Concentration of VOCs released from FOM and wild ancestors of *U. molinae*. (**B**) Concentration of VOCs released from FOM and wild ancestors of *U. molinae* subjected damages and (**C**) Concentration of VOCs released from each ecotype of *U. molinae* subjected damages. Different letters means significant differences according to ANOVA followed by Tukey test (*p* ≤ 0.05). * means significant differences according to the *t*-test.

Figure 1A shows a significant release of VOCs in wild relatives, with 624.6 ± 53.0 µg/cm^2^/day, compared to the FOM ecotypes, which only reached a release of 276.6 ± 45.0 µg/cm^2^/day. This is mainly supported by the release of VOCs from the wild relative ecotypes subjected to herbivory damage treatment, reaching a release of 439.3 ± 20.0 µg/cm^2^/day. This corresponds to 70.33% of the total VOCs released by wild relatives and 58.8% of the total release of the FOM ecotypes. In addition, this level is significantly higher than all FOM ecotypes for control treatment (76.8 ± 4.4 µg/cm^2^/day), mechanical damage (82.9 ± 21.7 µg/cm^2^/day), and herbivory damage (112.8 ± 17.4 µg/cm^2^/day), as observed in Figure 1B. A significant emission of VOCs by ecotype is observed for the treatment of herbivory, which is explained by the release of VOCs from the ecotypes 22-1 (192.7 ± 11.5 µg/cm^2^/day), 19-1 (145.6 ± 5.7 µg/cm^2^/day), and 23-2 (101.0 ± 2.7 µg/cm^2^/day), representing 43.86%, 33.14%, and 22.99%, respectively. Therefore, the differences found indicate a significant release of VOCs in wild relatives subjected to herbivory damage compared to FOM ecotypes (Figure 1C).

### 3.3. GLVs Related to the Domestication Degree

**Figure 2:**
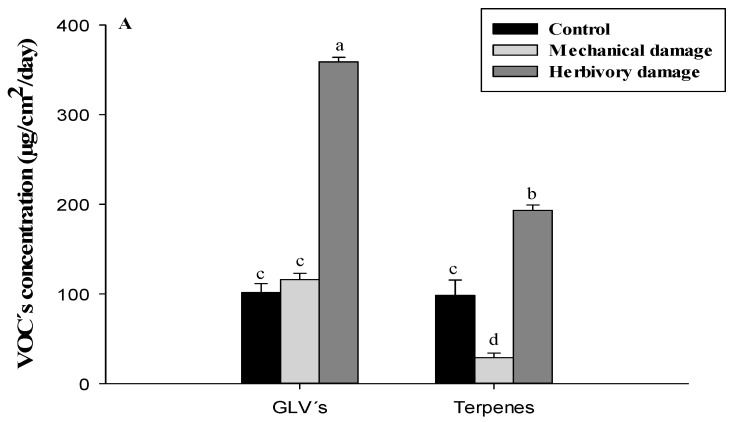

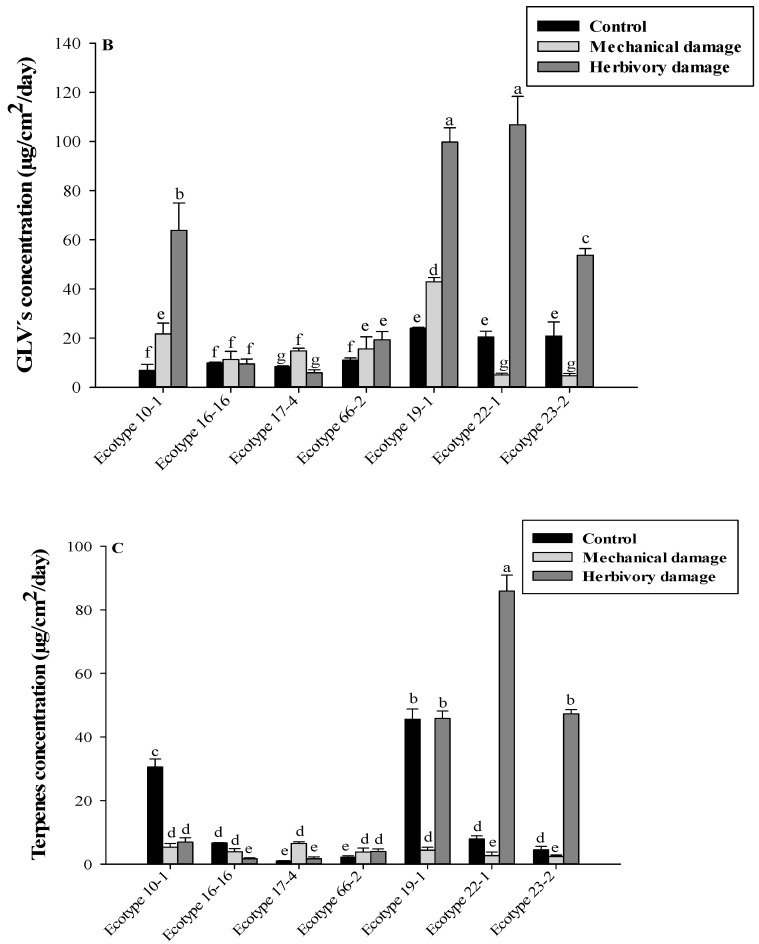
(**A**) concentration of VOCs released from *U. molinae* separated by GLVs and terpenes. (**B**) concentration of GLVs released from *U. molinae* and (**C**) concentration of terpenes released from *U. molinae.* Different letter means significant differences according to ANOVA followed by Tukey test (*p* ≤ 0.05).

Regarding the types of compounds released by murtilla plants (Figure 2A), the highest release rate was represented by GLVs, accounting for 39.81% of the total VOCs released, while terpenes only accounted for 21.43% of the total release of VOCs for herbivory damage treatment.

The release of GLVs was highest in ecotypes 22-1 (106.8 ± 11.5 µg/cm^2^/day) and 19-1 (99.8 ± 5.7 µg/cm^2^/day) for the herbivory damage treatment, accounting for 29.76% and 27.81% of the total release, respectively. These two wild relative ecotypes, along with the herbivory damage treatment, showed significantly higher release rates of GLVs compared to all four FOM ecotypes. Additionally, ecotype 23-2 (wild relative) showed a significantly higher release rate of GLVs than FOM ecotypes. Table 3 shows the analysis of individual compounds found by HPLC-DAD. Additionally, ecotype 23-2 (wild relative) also exhibited significantly higher release rates of GLVs than FOM ecotypes. The analysis of individual compounds found by HPLC-DAD is presented in Table 3, where 2-hexanone was significantly higher in FOM 10-1 and 66-2 for the control treatment with values of 0.8 ± 0.1 µg/cm^2^/day for both ecotypes. However, when the wild relative 19-1 was subjected to mechanical damage or herbivory treatment, there was an increase of almost 10-fold and 6-fold, respectively, in its concentration (0.4 ± 0.0 µg/cm^2^/day vs. 3.9 ± 0.6 µg/cm^2^/day and 2.3 ± 0.3 µg/cm^2^/day, respectively). Similarly, 3-hexanone, 2-hexanol, and 3-hexanol showed an increase of about four-, six-, and seven-fold, respectively, when subjected to mechanical damage treatment for the wild relative 19-1. When comparing the control with herbivory damage treatment, the wild relative 19-1 exhibited an increase of three-, six-, and six-fold for these compounds, respectively. Table 4 compares the wild relative 22-1 with their respective FOMs, where there was an increase in concentration for all seven ecotypes when subjected to herbivory damage treatment for the group of alcohols and ketones. Among these, the wild relative 22-1 showed the most significant increase, with 17.5-, 9.2-, 9.1-, and 5.5-fold increases for 2-hexanone, 3-hexanone, 2-hexanol, and 3-hexanol, respectively, compared to the control treatment. When comparing the wild relative 23-2 with their respective FOMs, it was observed that the wild relative 23-2 presented the greatest increases in each compound for the groups of compounds such as alcohols and ketones, monoterpenes, and esters, when subjected to herbivory damage treatment. The increases ranged from 6.2-fold for 3-hexanol to 53-fold for 3-hexanone compared to the control treatment.

### 3.4. Terpenes Related to the Domestication Degree

The release of terpenes (Figure 2C) was highest in ecotype 22-1, representing 44.46% of the total terpenes released for herbivory damage treatment, with a concentration of 85.9 ± 5.0 µg/cm^2^/day. In ecotypes 19-1 and 23-2, herbivory damage resulted in significantly higher terpene release compared to their respective offspring and to the total released from all four FOM ecotypes. However, the release of monoterpenes was generally reduced by mechanical and herbivory damage treatments, except for *β*-myrcene in the wild relative 19-1, which showed a 2.6-fold increase in release compared to the control treatment when subjected to herbivory damage. Additionally, the wild relative 19-1 had the highest concentration of the ester 2,4-D dimethyl acetophenone compared to all four FOM ecotypes, with values of 23.1 ± 2.7 µg/cm^2^/day, 21.1 ± 2.1 µg/cm^2^/day, and 82.5 ± 2.2 µg/cm^2^/day for control treatment, mechanical damage, and herbivory damage, respectively. In terms of sesquiterpenes, caryophyllene showed a 3.1-fold increase when subjected to mechanical damage in the wild relative 19-1, while *α*-caryophyllene was poorly detected in general for all ecotypes (wild relatives and FOM) and treatments. In relation to the release of monoterpenes, the wild relative 22-1 showed a significant increase in herbivory treatment of 7.6-, 26.0-, 11.0-, 38.2-, and 17.4-fold compared to the control treatment in pinene, sabinene, *β*-myrcene, 1,8 cineole, and limonene, respectively. For ester concentration, the wild relative 22-1 increased by about 4.6-fold when subjected to herbivory damage. A similar situation was observed in FOM 10-1, which increased its value by 11.8-fold in herbivory damage treatment. Regarding sesquiterpenes, there was an Increasing trend for the wild relative 22-1 when subjected to herbivory damage, reaching 1.8 ± 1.2 µg/cm^2^/day. Finally, the release of sesquiterpenes in the wild relative 23-2 when exposed to mechanical damage for both caryophyllene (1.7 ± 0.7 µg/cm^2^/day) and *α*-caryophyllene (0.3 ± 0.1 µg/cm^2^/day) was higher than in mechanical and control treatments (Table 5).

## 4. Discussion

According to the plant domestication defense theory, selective breeding or domestication in *U. molinae* has been linked to a reduction in defensive compounds that are useful for coping with insect pests [9,10,11,12,17,31,47,48]. Overall, our results related to the release of VOCs by *U. molinae* were consistent with the plant domestication defense theory, as we observed an increase in the emission of VOCs in plants that were subjected to herbivory damage compared to control plants. While Scheuermann et al. [14] reported different VOCs for murtilla, their research only evaluated volatile compounds for fruits without considering the influence of domestication or a comparison between wild relatives and FOM. However, Chacón-Fuentes et al. [15] reported VOCs for murtilla plants, such as 2-hexanone, α-pinene, 1,8-cineole, and *trans*-caryophyllene, which are in agreement with our results. In addition, the authors demonstrated that there was a reduction in these compounds in cultivated plants due to the domestication effect. They also showed that mechanical damage to murtilla plants increased the abundance of these VOCs, which contrasts with our findings.

Studies by Rodríguez-Saona et al. [30] regarding herbivory damage reported that the induction of VOCs due to gypsy moth (Lepidoptera: Erebidae) feeding was the main factor involved in alcohol and ester emissions for cranberries. Moreover, VOCs such as hexanol, myrcene, limonene, 1,8-cineole, and caryophyllene were influenced by gypsy moth feeding in cranberry plants. We found that mechanical damage did not significantly influence the emission of VOCs, in contrast to the herbivory damage treatment. This is notable given that VOCs such as monoterpenes or sesquiterpenes are well-known for acting as warning compounds released by plants to repel insect pests or attract natural enemies [16,17,18,19,20]. Furthermore, the abundance of these compounds typically increases when plants are subjected to external damage, such as mechanical or herbivory damage [15,21,22]. Thus, it is clear that the emission of VOCs can be influenced by various factors, and their role in plant defense mechanisms can vary depending on the type of damage or stress that the plant experiences. For instance, Naranjo-Guevara et al. [27] reported that undamaged wild relatives and domesticated maize did not show a release of *β*-myrcene; however, when they were exposed to *Spodoptera frugiperda* (Lepidoptera: Noctuidae) feeding, *β*-myrcene reached 0.1 and 0.05 ng/12 h/plant in the wild relative and modern maize, respectively. In addition, reports by deLange et al. [23] showed that the number of the parasitoid *Cotesia marginiventris* (Hymenoptera: Braconidae) increased twofold in wild relatives when they were exposed to *Spodoptera littoralis* (Lepidoptera: Noctuidae) feeding compared to cultivated maize. To date, there is no literature about herbivory damage and its relation to the emission of VOCs in *U. molinae*.

The effect of mechanical damage on the VOCs emitted from *U. molinae* plants that underwent a domestication process was studied by Chacón-Fuentes et al. [15], who indicated that mechanical damage increased the amount of 2-hexanone and 1,8-cineole, as well as the production of α-pinene. Similarly, Zhang et al. [49] reported an increase in the concentration of α-pinene and limonene in persimmon trees from non-detected to 36.60 and 36.10 µg/h, respectively, when Japanese wax scale (Hemiptera: Coccidae) damage was induced. Harmel et al. [50] also reported an increase in the emission of the monoterpene linalool by 7% in potato plants infested with the aphid *M. persicae* compared to mechanical damage and control. Salamanca et al. [51] reported an increase in limonene from non-detected to 0.71 µg/h in rose plants when they were infested with the aphid *Macrosiphum euphorbiae* (Hemiptera: Aphididae), which is consistent with our results showing an increase in VOC release in plants subjected to herbivory damage. However, studies by Gouinguene et al. [52] reported no significant differences in the emissions of β-myrcene and *β*-caryophyllene in the maize wild relative *Zea perennis* and the modern cultivar, suggesting relevant variation in VOC release. *U. molinae* plants are undergoing an incipient domestication process through selective breeding, and thus the gap between wild relatives and cultivated plants is still narrow.

In this context, murtilla is a shrub that naturally develops and is able to grow in different locations and under several stresses [2]. Therefore, *U. molinae* plants subjected to mechanical damage may not identify this damage as a threat. However, when herbivory damage is provoked, plants are able to identify proteins or elements in the insect saliva as a real threat, as reported by several authors [53,54]. For instance, Alborn et al. [53] indicated that a protein in *Spodoptera exigua* (Lepidoptera: Noctuidae) saliva elicited the induction of maize VOCs in response to the insect attack. In general, the theory of plant domestication defense is applicable for the emission of VOCs in murtilla plants, and the domestication effect is also noticeable when wild relatives are compared with their respective FOMs. The results presented here reveal a considerable increase in the VOCs released when herbivory damage was provoked by aphids. Wild relatives 19-1, 22-1, and 23-2 were highlighted ecotypes in relation to VOC emission, particularly with the 2,4-dimethyl acetophenone concentration. Therefore, this study demonstrates how plant defense in murtilla is diminished by the effect of domestication, even at an incipient level, as is the case with FOM. It also generates knowledge about the composition of VOCs present in murtilla plants and delivers a vision regarding the volatile secondary metabolism that will allow for focusing or directing the genetic efforts to obtain more balanced plants in the next stages of murtilla domestication. Finally, an important next step is to determine which of the VOCs generated by murtilla are essential in the attractive behavior of natural enemies or pests, or even related to the generation of food-grade technologies.

## 5. Conclusions

Based on the results presented in this study, it can be concluded that wild relatives of *Ugni molinae* release significantly higher amounts of VOCs, particularly GLVs, in response to herbivory damage compared to cultivated ecotypes. This suggests that domestication may have reduced the ability of cultivated ecotypes to produce and release VOCs in response to herbivore attack. The identification of specific compounds that contribute to this response, such as hexanone, hexanol, pinene, and caryophyllene, provides valuable information for further studies aiming to develop new methods for pest management and plant breeding strategies aimed at enhancing plant resistance to herbivores.

## Figures and Tables

**Table 1:** Origin of first offspring of murtilla and its wild relatives involved in the breeding.

First Reciprocal Crosses (FOM)	Wild Relatives
Ecotype 10-1	Ecotypes 22-1 × ecotype 19-1
Ecotype 16-16	Ecotypes 19-1 × ecotype 22-1
Ecotype 17-4	Ecotypes 23-2 × ecotype 22-1
Ecotype 66-2	Ecotypes 23-2 × ecotype 19-1

**Table 2:** Kovats indices for all VOCs identified in both cultivated ecotypes and wild relatives of damaged and undamaged murtilla detected by GC-MS.

RT (min)	Group	Compound	KI_exp_	KI_Lib_	Reference
4.37	*Ketones*	2-Hexanone	-	750	[37] *
4.44		3-Hexanone	-	786	[38] *
4.71	*Alcohols*	3-Hexanol	-	797	[39] *
4.83		2-Hexanol	-	803	[40] *
8.28	*Monoterpenes*	Pinene	929	931	[41] *
9.40		Sabinene	969	969	[41] *
9.94		*β*-Myrcene	986	988	[41] *
10.87		1,8 Cineole	1018	1015	[42] *
10.99		Limonene	1022	1025	[43] *
17.29	*Ester*	2,4-Dimethyl acetophenone	1240	-	[44] *
21.79	*Sesquiterpenes*	Caryophyllene	1409	1414	[45] *
22.68		*α*-Caryophyllene	1446	1442	[46] *

KI_exp_: Represents the experimental Kovats indices, KI_lib_: represents the library Kovats indices, RT: represents the retention time in minutes. An asterisk (*) indicates a compound that was compared to a commercial standard.

**Table 3:** Comparison of VOCs released among both undamaged and herbivory and mechanically damaged wild relative 19-1 and their cultivated ecotypes. Different lowercase letters for each damage treatment means significant differences according to ANOVA followed by Tukey test. Absence of uppercase letters for each individual compound means no significant difference among damage treatments to ANOVA followed by Tukey test. ND = No detected.

Groups		Control				Mechanical Damage				Herbivory Damage			
	Compounds/ Ecotypes	19-1	10-1	16-16	66-2	19-1	10-1	16-16	66-2	19-1	10-1	16-16	66-2
*Alcohols and cetones*	2-Hexanone	0.4 ± 0.0 ^b^	0.8 ± 0.1 ^a^	0.3 ± 0.0 ^b^	0.8 ± 0.1 ^a^	3.9 ± 0.6 ^a^	1.7 ± 0.2 ^b^	0.7 ± 0.0 ^c^	2.1 ± 0.3 ^b^	2.3 ± 0.3 ^a^	2.1 ± 0.3 ^a^	1.7 ± 0.6 ^a^	1.9 ± 0.2 ^a^
	3-Hexanone	0.2 ± 0.0 ^b^	0.4 ± 0.0 ^a^	0.5 ± 0.0 ^a^	0.5 ± 0.1 ^a^	4.2 ± 0.1 ^a^	2.4 ± 0.4 ^b^	0.4 ± 0.0 ^c^	2.5 ± 0.1 ^b^	3.0 ± 0.5 ^b^	1.8 ± 0.2 ^c^	2.7 ± 0.1 ^b^	9.7 ± 1.8 ^a^
	3-Hexanol	0.1 ± 0.0 ^c^	0.3 ± 0.0 ^b^	0.5 ± 0.0 ^a^	0.6 ± 0.0 ^a^	6.0 ± 0.6 ^a^	1.9 ± 0.2 ^b^	1.1 ± 0.1 ^c^	2.1 ± 0.4 ^b^	5.9 ± 0.7 ^a^	3.2 ± 0.4 ^b^	1.7 ± 0.3 ^c^	3.6 ± 0.7 ^b^
	2-Hexanol	0.2 ± 0.0 ^c^	0.9 ± 0.1 ^b^	1.2 ± 0.1 ^b^	0.8 ± 0.0 ^b^	7.7 ± 1.9 ^a^	4.3 ± 0.7 ^b^	1.1 ± 0.1 ^d^	2.8 ± 0.5 ^c^	6.2 ± 0.6 ^a^	3.2 ± 0.3 ^b^	3.4 ± 0.0 ^b^	3.9 ± 0.6 ^b^
*Monoterpenes*	Pinene	15.9 ± 2.4 ^a^	10.2 ± 1.1 ^b^	3.4 ± 0.3 ^c^	1.1 ± 0.2 ^d^	1.6 ± 0.0 ^a^	1.0 ± 0.1 ^b^	1.5 ± 0.2 ^a^	0.9 ± 0.1 ^b^	12.6 ± 1.2 ^a^	2.3 ± 0.4 ^c^	0.9 ± 0.1 ^d^	3.3 ± 0.1 ^b^
	Sabinene	4.7 ± 0.2 ^b^	7.0 ± 0.4 ^a^	0.2 ± 0.0 ^c^	0.3 ± 0.0 ^c^	0.6 ± 0.0 ^b^	1.5 ± 0.4 ^a^	1.5 ± 0.2 ^a^	0.5 ± 0.0 ^b^	15.3 ± 0.2 ^a^	3.4 ± 0.9 ^b^	0.1 ± 0.0 ^c^	0.1 ± 0.0 ^c^
	β-Myrcene	2.8 ± 0.3 ^b^	5.3 ± 0.3 ^a^	1.0 ± 0.1 ^c^	0.2 ± 0.0 ^d^	ND	0.4 ± 0.0 ^a^	ND	0.5 ± 0.0 ^a^	7.5 ± 0.0 ^a^	ND	0.1 ± 0.0 ^b^	0.1 ± 0.0 ^b^
	1,8 Cineole	7.9 ± 0.5 ^a^	2.6 ± 0.6 ^b^	0.3 ± 0.0 ^c^	0.2 ± 0.0 ^c^	0.2 ± 0.0 ^b^	0.3 ± 0.0 ^a^	0.3 ± 0.0 ^a^	0.4 ± 0.0 ^a^	5.1 ± 0.0 ^a^	0.1 ± 0.0 ^b^	0.2 ± 0.0 ^b^	0.1 ± 0.0 ^b^
	Limonene	13.7 ± 1.4 ^a^	10.0 ± 1.1 ^a^	0.5 ± 0.0 ^b^	0.2 ± 0.0 ^c^	0.1 ± 0.0 ^d^	1.4 ± 0.2 ^a^	0.6 ± 0.0 ^c^	0.9 ± 0.0 ^b^	5.3 ± 0.3 ^a^	0.7 ± 0.0 ^b^	0.4 ± 0.0 ^c^	0.2 ± 0.0 ^d^
*Esters*	2,4-Dimethyl acetophenone	23.1 ± 2.7 ^a^	4.5 ± 0.3 ^c^	7.4 ± 1.2 ^b^	8.4 ± 1.4 ^b^	21.1 ± 2.1 ^a^	11.4 ± 1.7 ^b^	8.0 ± 1.1 ^c^	6.1 ± 0.6 ^c^	82.5 ± 2.2 ^a^	53.5 ± 8.4 ^b^	ND	0.2 ± 0.0 ^c^
*Sesquiterpenes*	Caryophyllene	0.6 ± 0.0 ^b^	0.4 ± 0.0 ^b^	1.2 ± 0.1 ^a^	0.2 ± 0.0 ^c^	1.9 ± 0.2 ^b^	0.6 ± 0.0 ^a^	ND	0.5 ± 0.0 ^a^	ND	0.2 ± 0.0 ^a^	ND	0.1 ± 0.0 ^a^
	α-Caryophyllene	ND ^b^	ND ^b^	0.1 ± 0.0 ^a^	ND ^b^	ND	0.1 ± 0.0 ^a^	ND	0.1 ± 0.0 ^a^	ND	0.2 ± 0.0 ^a^	ND	0.1 ± 0.0 ^a^

**Table 4:** Comparison of VOCs released among undamaged and herbivory and mechanically damaged wild relative 22-1 and their cultivated ecotypes. Different lowercase letters for each damage treatment means significant differences according to ANOVA followed by Tukey test. Absence of uppercase letters for each individual compounds means no significant differences among damage treatments according to ANOVA followed by Tukey test. ND = No detected.

Groups		Control				Mechanical Damage				Herbivory Damage			
	Compounds/ Ecotypes	22-1	10-1	16-16	17-4	22-1	10-1	16-16	17-4	22-1	10-1	16-16	17-4
*Alcohols and cetones*	2-Hexanone	0.4 ± 0.0 ^b^	0.8 ± 0.1 ^a^	0.3 ± 0.0 ^c^	ND	0.4 ± 0.3 ^c^	1.7 ± 0.2 ^a^	0.7 ± 0.0 ^b^	2.1 ± 0.6 ^a^	7.0 ± 1.1 ^a^	2.1 ± 0.3 ^b^	1.7 ± 0.6 ^c^	0.5 ± 0.2 ^d^
	3-Hexanone	0.8 ± 0.5 ^a^	0.4 ± 0.0 ^c^	0.5 ± 0.0 ^b^	0.2 ± 0.2 ^d^	0.9 ± 0.6 ^b^	2.4 ± 0.4 ^a^	0.4 ± 0.0 ^c^	2.8 ± 1.3 ^a^	7.4 ± 1.7 ^a^	1.8 ± 0.2 ^c^	2.7 ± 0.1 ^b^	0.4 ± 0.2 ^d^
	3-Hexanol	0.7 ± 0.1 ^a^	0.3 ± 0.0 ^c^	0.5 ± 0.0 ^b^	ND	0.8 ± 0.5 ^d^	1.9 ± 0.2 ^b^	1.1 ± 0.1 ^c^	3.7 ± 1.3 ^a^	6.4 ± 0.7 ^a^	3.2 ± 0.4 ^b^	1.7 ± 0.3 ^c^	1.0 ± 0.6 ^c^
	2-Hexanol	0.8 ± 0.1 ^c^	0.9 ± 0.1 ^b^	1.2 ± 0.1 ^a^	0.3 ± 0.3 ^d^	1.9 ± 0.9 ^c^	4.3 ± 0.7 ^b^	1.1 ± 0.1 ^d^	6.2 ± 2.2 ^a^	4.4 ± 0.2 ^a^	3.2 ± 0.3 ^b^	3.4 ± 0.0 ^b^	0.6 ± 0.3 ^c^
*Monoterpenes*	Pinene	3.9 ± 0.5 ^b^	10.2 ± 1.1 ^a^	3.4 ± 0.3 ^b^	0.7 ± 0.3 ^c^	0.3 ± 0.2 ^d^	1.0 ± 0.1 ^c^	1.5 ± 0.2 ^b^	3.7 ± 3.0 ^a^	29.9 ± 6.6 ^a^	2.3 ± 0.4 ^b^	0.9 ± 0.1 ^c^	1.2 ± 0.7 ^c^
	Sabinene	0.5 ± 0.3 ^b^	7.0 ± 0.4 ^a^	0.2 ± 0.0 ^c^	0.1 ± 0.0 ^c^	0.2 ± 0.2 ^b^	1.5 ± 0.4 ^a^	1.5 ± 0.2 ^a^	0.5 ± 0.4 ^b^	13.0 ± 1.8 ^a^	3.4 ± 0.9 ^b^	0.1 ± 0.0 ^c^	0.1 ± 0.1 ^c^
	β-Myrcene	1.2 ± 0.7 ^b^	5.3 ± 0.3 ^a^	1.0 ± 0.1 ^b^	ND	0.1 ± 0.0 ^c^	0.4 ± 0.0 ^b^	ND	0.9 ± 0.6 ^a^	13.2 ± 2.0 ^a^	ND	0.1 ± 0.0 ^b^	ND
	1,8 Cineole	0.4 ± 0.3 ^b^	2.6 ± 0.6 ^a^	0.3 ± 0.0 ^b^	0.1 ± 0.1 ^b^	0.1 ± 0.0 ^b^	0.3 ± 0.0 ^a^	0.3 ± 0.0 ^a^	0.1 ± 0.1 ^b^	15.3 ± 0.2 ^a^	0.1 ± 0.0 ^b^	0.2 ± 0.0 ^b^	ND
	Limonene	0.7 ± 0.4 ^b^	10.0 ± 1.1 ^a^	0.5 ± 0.0 ^b^	0.1 ± 0.1 ^c^	1.0 ± 0.9 ^b^	1.4 ± 0.2 ^a^	0.6 ± 0.0 ^c^	0.4 ± 0.2 ^c^	12.2 ± 1.9 ^a^	0.7 ± 0.0 ^b^	0.4 ± 0.0 ^c^	0.3 ± 0.2 ^c^
*Esters*	2,4-Dimethyl acetophenone	17.7 ± 3.0 ^a^	4.5 ± 0.3 ^c^	7.4 ± 1.2 ^b^	7.9 ± 9.1 ^b^	5.8 ± 3.2 ^c^	11.4 ± 1.7 ^a^	8.0 ± 1.1 ^b^	13.3 ± 9.2 ^a^	81.6 ± 1.3 ^a^	53.5 ± 8.4 ^b^	ND	3.4 ± 2.1 ^c^
*Sesquiterpenes*	Caryophyllene	1.2 ± 0.8 ^a^	0.4 ± 0.0 ^b^	1.2 ± 0.1 ^a^	ND	0.9 ± 0.8 ^a^	0.6 ± 0.0 ^b^	ND	0.9 ± 0.8 ^a^	1.8 ± 1.2 ^a^	0.2 ± 0.0 ^b^	ND	ND
	α-Caryophyllene	ND	ND	0.1 ± 0.0 ^d^	ND	0.1 ± 0.1 ^a^	0.1 ± 0.0 ^a^	ND	ND	0.5 ± 0.3 ^a^	0.2 ± 0.0 ^b^	ND	0.1 ± 0.1 ^c^

**Table 5:** Comparison of VOCs released among wild relative 23-1 and their cultivated ecotypes on undamaged and herbivory and mechanical damages. Absence of lowercase letters for each damage treatment means significant differences according to ANOVA followed by Tukey test. Absence of uppercase letters for each individual compound means no significant differences among damage treatments according to ANOVA followed by Tukey test. ND = No detected.

Groups		Control			Mechanical Damage			Herbivory Damage		
	Compounds/Ecotypes	23-2	17-4	66-2	23-2	17-4	66-2	23-2	17-4	66-2
*Alcohols and cetones*	2-Hexanone	0.1 ± 0.0 ^b^	ND	0.8 ± 0.1 ^a^	0.5 ± 0.5 ^b^	2.1 ± 0.6 ^a^	2.1 ± 0.3 ^a^	5.3 ± 0.1 ^a^	0.5 ± 0.2 ^c^	1.9 ± 0.2 ^b^
	3-Hexanone	1.9 ± 0.2 ^a^	0.2 ± 0.2 ^c^	0.5 ± 0.1 ^b^	0.7 ± 0.5 ^b^	2.8 ± 1.3 ^a^	2.5 ± 0.1 ^a^	5.7 ± 0.3 ^b^	0.4 ± 0.2 ^c^	9.7 ± 1.8 ^a^
	3-Hexanol	0.7 ± 0.1 ^a^	ND	0.6 ± 0.0 ^a^	0.5 ± 0.3 ^b^	3.7 ± 1.3 ^a^	2.1 ± 0.4 ^a^	4.4 ± 0.2 ^a^	1.0 ± 0.6 ^c^	3.6 ± 0.7 ^b^
	2-Hexanol	1.9 ± 0.7 ^a^	0.3 ± 0.3 ^c^	0.8 ± 0.0 ^b^	1.0 ± 0.5 ^b^	6.2 ± 2.2 ^a^	2.8 ± 0.5 ^b^	20.2 ± 19.0 ^a^	0.6 ± 0.3 ^c^	3.9 ± 0.6 ^b^
*Monoterpenes*	Pinene	1.2 ± 0.8 ^a^	0.7 ± 0.3 ^b^	1.1 ± 0.2 ^a^	0.1 ± 0.1 ^c^	3.7 ± 3.0 ^a^	0.9 ± 0.1 ^b^	13.8 ± 3.3 ^a^	1.2 ± 0.7 ^c^	3.3 ± 0.1 ^b^
	Sabinene	1.3 ± 0.9 ^a^	0.1 ± 0.0 ^b^	0.3 ± 0.0 ^b^	0.1 ± 0.1 ^b^	0.5 ± 0.4 ^a^	0.5 ± 0.0 ^a^	10.6 ± 0.5 ^a^	0.1 ± 0.1 ^b^	0.1 ± 0.0 ^b^
	β-Myrcene	0.6 ± 0.5 ^a^	ND	0.2 ± 0.0 ^b^	0.1 ± 0.1 ^c^	0.9 ± 0.6 ^a^	0.5 ± 0.0 ^b^	15.2 ± 0.1 ^a^	ND	0.1 ± 0.0 ^b^
	1,8 Cineole	0.3 ± 0.2 ^a^	0.1 ± 0.1 ^a^	0.2 ± 0.0 ^a^	0.1 ± 0.1 ^b^	0.1 ± 0.1 ^b^	0.4 ± 0.0 ^a^	7.5 ± 0.0 ^a^	ND	0.1 ± 0.0 ^b^
	Limonene	1.1 ± 0.8 ^a^	0.1 ± 0.1 ^b^	0.2 ± 0.0 ^b^	ND	0.4 ± 0.2 ^b^	0.9 ± 0.0 ^a^	0.1 ± 0.1 ^a^	0.3 ± 0.2 ^a^	0.2 ± 0.0 ^a^
*Esters*	2,4-Dimethyl acetophenone	16.2 ± 3.0 ^a^	7.9 ± 9.1 ^c^	8.4 ± 1.4 ^b^	2.3 ± 1.6 ^c^	13.3 ± 9.2 ^a^	6.1 ± 0.6 ^b^	18.1 ± 2.8 ^a^	3.4 ± 2.1 ^b^	0.2 ± 0.0 ^c^
*Sesquiterpenes*	Caryophyllene	ND	ND	0.2 ± 0.0 ^d^	1.7 ± 0.7 ^a^	0.9 ± 0.8 ^b^	0.5 ± 0.0 ^b^	ND	ND	0.1 ± 0.0 ^a^
	α-Caryophyllene	ND	ND	ND	0.3 ± 0.1 ^a^	ND	0.1 ± 0.0 ^b^	0.1 ± 0.1 ^a^	0.1 ± 0.1 ^a^	0.1 ± 0.0 ^a^

## Data Availability

The data presented in this study are available on request from the corresponding author. The data are not publicly available due to privacy.
